# Genetic Diversity of Cowpea (*Vigna unguiculata* (L.) Walp.) Accession in Kenya Gene Bank Based on Simple Sequence Repeat Markers

**DOI:** 10.1155/2016/8956412

**Published:** 2016-11-24

**Authors:** Emily N. Wamalwa, John Muoma, Clabe Wekesa

**Affiliations:** ^1^Department of Biological Sciences, Masinde Muliro University of Science and Technology, P.O. Box 190-50100, Kakamega, Kenya; ^2^Department of Biochemistry and Biotechnology, Kenyatta University, P.O. Box 43844-00100, Nairobi, Kenya

## Abstract

Increased agricultural production is an urgent issue. Projected global population is 9 million people by mid of this century. Estimation projects death of 1 million people for lack of food quality (micronutrient deficit) and quantity (protein deficit). Majority of these people will be living in developing countries. Other global challenges include shrinking cultivable lands, salinity, and flooding due to climate changes, new emerging pathogens, and pests. These affect crop production. Furthermore, they are major threats to crop genetic resources and food security. Genetic diversity in cultivated crops indicates gene pool richness. It is the greatest resource for plant breeders to select lines that enhance food security. This study was conducted by Masinde Muliro University to evaluate genetic diversity in 19 cowpea accessions from Kenya national gene bank. Accessions clustered into two major groups. High divergence was observed between accessions from Ethiopia and Australia and those from Western Kenya. Upper Volta accessions were closely related to those from Western Kenya. Low variation was observed between accessions from Eastern and Rift Valley than those from Western and Coastal regions of Kenya. Diversity obtained in this study can further be exploited for the improvement of cowpea in Kenya as a measure of food security.

## 1. Introduction

Cowpea,* Vigna unguiculata *(L.) Walp. (2*n* = 22), is one of the most ancient human food sources [1]. It is one of the most important legume crops in the world and it is a major food crop in Africa, Latin America, and India because of its high protein content [2, 3]. As a result, cowpea is referred to as a poor man's meat [4]. Cowpea is primarily a self-pollinating crop and its genetic base is considered to be narrow [5–7].

The world's cowpea production as at 2013 was estimated at 5,718,144.66 tonnes of which 5,421,561 tonnes were from Africa, with East Africa contributing 532,901 tonnes [8]. In Kenya, yields remain extremely low, ranging from 150 to 500 kg/ha which is attributed to abiotic and biotic stresses, lack of high yielding cultivars, and poor crop management practices [9]. The area under cowpea in Kenya is estimated at 1800 ha excluding the area under the crop in home gardens [10].

Traditionally, diversity in cowpea is estimated by measuring variation in phenotypic or qualitative traits such as flower colour, growth habit, or quantitative agronomic traits such as yield potential and stress tolerance [11] that do not necessarily reflect real genetic relationships [12]. Furthermore, the expression of quantitative traits is subject to strong environmental influence and therefore limits knowledge of the germplasm structure for development of hybrids with specific ecological adaptations [11]. DNA markers are the most popular and widely used techniques to differentiate among genotypes at species and subspecies level [13]. Comparative studies in plants have shown that simple sequence repeat (SSR) markers, which are single locus markers with multiple alleles, are more valuable and provide an effective means for discriminating between genotypes [14, 15]. This study characterized 19 cowpea accessions from different sources that had been preserved at the national gene bank of Kenya using two SSR markers (Table 2) that had highest polymorphic amplification of both local and inbred lines of cowpea as reported by Badiane et al. [16].

## 2. Materials and Methods

### 2.1. DNA Extraction

Seedlings of each cowpea accession (Table 1) were grown in pots of sterile soil in a greenhouse with 3 plants per accession. Leaf samples were purposively sampled from three plants per accession from 15-day-old seedlings [16] and frozen in liquid nitrogen and genomic DNA extracted according to the prescribed protocol of the DNeasy Plant Mini Kit (Qiagen).

### 2.2. PCR Amplification of DNA and Electrophoresis

PCR was carried out in 0.2 mL tubes with a reaction volume of 25 *μ*L containing 2.5 *μ*L 10x PCR buffer, 1 *μ*L of both primers, 1 mM of each dNTPs, 0.5 U Taq DNA polymerase, and 50 ng DNA. The tubes were placed in an Eppendorf Mastercycler Gradient thermocycler programmed for initial denaturation at 94°C for 1 minute, followed by 35 cycles for 30 seconds at 94°C, 30 seconds at 55°C, 1 minute at 75°C, and final extension for 10 minutes at 72°C.

**Figure 1:**
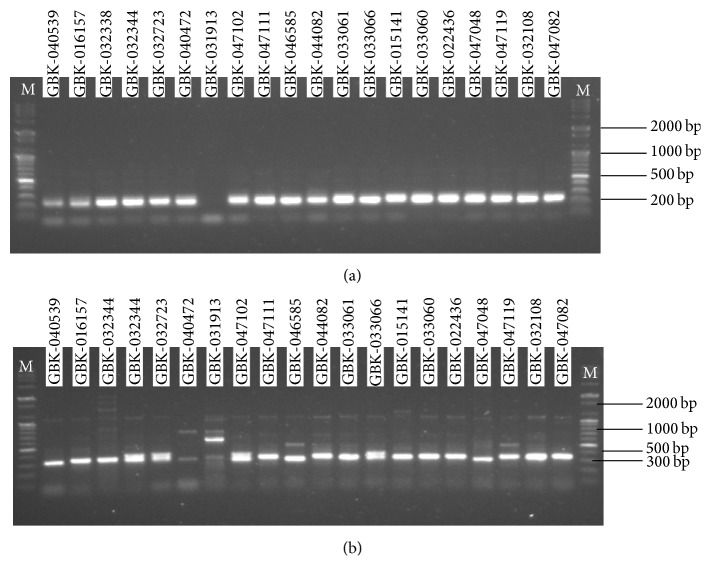
(a) PCR products of the 20 accessions of cowpea amplified using SSR primer 6652: M indicates the ladder. (b) PCR products of the 20 accessions of cowpea amplified using SSR primer 6540: M indicates the ladder.

The PCR products were resolved on a agarose gel (1%) using 0.5x TBE containing 1 mg/mL ethidium bromide with a vertical electrophoresis apparatus. The gel was photographed using Alphaimager 2200 under UV transilluminator (Figure 1). The resolved products were extracted from the gel and purified using the Qiagen DNA purification kit according to the prescribed protocol. DNA quantification was done by using a DNA NanoDrop 2000/2000c Spectrophotometer.

## 3. Phylogenetic Analysis 

The sequences obtained were first edited by BioEdit version (version 7) and then nucleotide alignments were generated using ClustalW software. The evolutionary history was inferred using the Neighbor-Joining method. Analyses were conducted using the Jukes-Cantor model [17]. The analysis involved 19 nucleotide sequences. Codon positions included were the 1st, 2nd, 3rd, and noncoding positions. All ambiguous positions were removed for each sequence pair. Evolutionary analyses were conducted in MEGA6. The confidence of the branches was measured by bootstrapping with 1,000 replicates [18].

## 4. Results

### 4.1. PCR Products

Nineteen cowpea accessions gave PCR products of ~300 bp (Figure 1(b)) for the SSR-6540 marker, while SSR-6652 gave PCR products of ~200 bp (Figure 1(a)) and these were not able to give any significant similarity from the NCBI and hence were not considered useful for this study.

### 4.2. Phylogenetic Analysis

**Figure 2:**
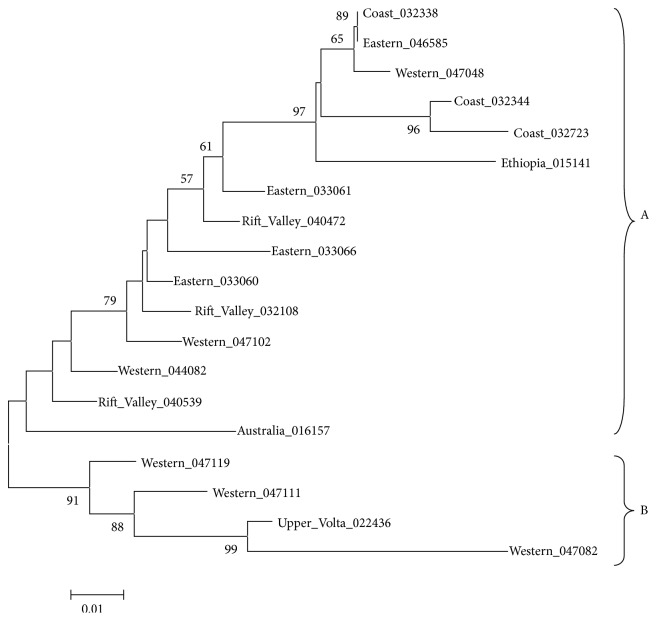
Phylogenetic tree of cowpea varieties: varieties having similar distances are genetically similar.

These accessions were clustered into two main groups: A and B, as indicated in Figure 2. Cluster A is comprised of 16 accessions most diverged that form seven subclusters with bootstrap support of 79 (Eastern_033066, Rift_Valley_032108, Eastern_033060, and Western_047102), 59 (Rift_Valley_040472), 62 (Eastern_033061), 96 (Ethiopia_015141), 98 (Coast_032344 and Coast_032373), 63 (Western_047048), and 88 (Eastern_046585, Coast_032338). Three accessions in this cluster (Western_044082, Rift_Valley_040539, and Australia_016157) had their bootstrap less than 50; hence, their branches were not reliable.

Cluster B is comprised of four accessions that exhibit moderate level of divergence, forming four subclusters with bootstrap support of 91 (Western_047119), 88 (Western_047111), and 99 (Western_047082 and Upper_Volta_022436).

## 5. Discussion

Genetic diversity is the extent to which material differs within a group of plants [19]. The low genetic variability in the cowpea accessions used in this study is consistent with the findings of previous studies due to the fact that a single domestication event is involved in the origin of this crop [7, 9, 20–24].

The low genetic divergence observed in this study is in agreement with the findings of Padulosi and Ng [25], who attributed it to the self-pollination nature of this crop. Given that the accessions were from different regions, it could also indicate high-gene flow within regions and limited time for significant genetic differentiation along geographical lines as indicated by Karuma et al. 2008 [9]. Highest levels of divergence between the accession from Western Kenya, Australia, and Ethiopia could be attributed to the fact that the accessions have been popularly cultivated in the respective regions over time giving enough time for significant genetic differentiation along geographical lines [9]. At the same time, it could indicate genetic evidence of cowpea being a very diverse taxon as reported by Huynh et al. 2013 [26]. This therefore would mean that the studied germplasm from Australia and Ethiopia has some amount of diversity that can be used for cowpea improvement in Western Kenya. In the same manner, it can be argued that Upper_Volta_022436 has some amount of diversity that can be used for improvement of cowpea at the Rift Valley and Eastern and Coastal region of Kenya. Eastern Kenya constitutes 85% of the total production of cowpea in Kenya [27]. The comparison of the genetic distances between accessions from Western Kenya (Western_047111, Western_047102, Western_044082, Western_047119, Western_047048, and Western_047082) to those from Eastern Kenya revealed a closer genetic relationship with Eastern_046585 than all other accessions from the same region (Eastern_033060, Eastern_033061, and Eastern_033066). It could be argued that these accessions could have common origin with accession Eastern_046585 and the observed variability is attributed to hybridization as indicated by Adewale et al. [28].

Similarly, two Western Kenya accessions (Western_047102 and Western_044082) exhibited a closer relationship with Rift Valley_032108. The low genetic distances observed between these accessions could possibly reflect the initial bottleneck during domestication maintained by the inherent self-pollination mechanism of cowpea crop [7]. On the contrary higher genetic distance observed between Western_047082 and Rift Valley_040539 and between Western_047048 and both Rift_Valley_040539 and Rift Valley_040472 could possibly indicate that the diversity in these accessions could be used for the improvement of cowpea crop in these two regions [26].

On the other hand, the genetic distances of the accessions from Western Kenya to the accessions from Australia, Ethiopia, and Upper Volta revealed that there was greater divergence between Western_047082 and the accession from Australia and Ethiopia, but this accession was genetically closely related with the accession from Upper Volta which is in agreement with Ba et al. [23]. This could mean that these two accessions (Western_047082 and Upper_Volta_022436) are actually the same accession, despite being collected from different regions. This study has also observed that there is higher diversity between Australia_016157 and the accessions from the Coastal region of Kenya which could be exploited to improve the cowpea germplasm in the Coastal region of Kenya. This study has proved that although the genetic base of cowpea is narrow, there exists some level of diversity between cowpea accessions in the Kenya gene bank that can be exploited for the improvement of cowpea crop as a measure for food security in Kenya.

## Figures and Tables

**Table 1:** List of the names of all 19 cowpea accessions used in this study and their geographic distributions (locations).

Sample number	Accession name	Genebank accession number	District and locality of collection	Latitude	Longitude	Date of collection
1	Rift Valley 040539	GBK-040539	Turkana; Nadoto	2.7333°N	35.11667°E	18.9.1994
2	Australia 016157	GBK-016157	Uasin Gishu	—	—	5.1.1989
3	Coast 032338	GBK-032338	Kwale; Mwachanda	—	—	11.2.1992
4	Coast 032344	GBK-032344	Kwale; Marenje village	4.46167°S	39.12833°E	11.3.1992
5	Coast 032723	GBK-032723	—	—	—	1.1.1976
6	Rift Valley 040472	GBK-040472	Kabarnet	—	—	9.2.1994
7	Coast 031913	GBK-031913	Busia	0.45694°N	34.191389°E	7.10.1992
8	Western 047102	GBK-047102	Kakamega; Bunyala East	0.44172°N	034.68136°E	22.11.2004
9	Western 047111	GBK-047111	Vihiga; Mudete	0.11785°N	034.76527°E	24.11.2004
10	Eastern 046585	GBK-046585	Mwingi; Nzelune-Makilungi	1.284167°N	38.258611°E	29.8.2003
11	Western 044082	GBK-044082	Meru; Nkubu market	0.066667°S	37.666667°E	2.11.1997
12	Eastern 033061	GBK-033066	Embu; Embu research station	3.508889°S	37.454722°E	1.12.1992
13	Eastern 033066	GBK-033061	Embu; Embu research station	3.508889°S	37.454722°E	1.12.1992
14	Ethiopia 015141	GBK-015141	Siaya; Kigilo	—	—	5.1.1989
15	Eastern 033060	GBK-033060	Embu; Embu research station	3.508889°S	37.454722°E	1.12.1992
16	Upper Volta 022436	GBK-022436	Kilifi	—	—	10.1.1975
17	Western 047048	GBK-047048	Busia; Ageng'a	0.22152°N	034.08540°E	19.11.2004
18	Western 047119	GBK-047119	Vihiga; Serem Tiriki East	0.07745°N	034.8548°E	24.11.2004
19	Rift Valley 032108	GBK-032108	Nandi; Kaptumo location	0.067500°N	35.067500°E	27.8.1992
20	Western 047082	GBK-047082	Busia; Bumala	0.30394°N	034.20103°E	19.11.2004

Data obtained from the national gene bank of Kenya.

**Table 2:** Primer sequences used.

Primer code	Primer sequence 5′ to 3′
SSR-6540	5′-GGACATTTAGGATTGGGTGG-3′
	5′-CCATAGGTTAAACTTATTGTACTC-3′
SSR-6652	5′-CAAAATTCCACGGTCACC-3′
	5′-CGGGACTTGAGGTAGCGCG-3′
